# Oral medications for the treatment of postural orthostatic tachycardia syndrome; a systematic review of studies before and during the COVID-19 pandemic

**DOI:** 10.3389/fneur.2024.1515486

**Published:** 2025-01-15

**Authors:** Benjamin C. Pierson, Kyle Apilado, M. Alaric Franzos, Rhonda Allard, James D. Mancuso, David Tribble, David Saunders, Tracey Perez Koehlmoos

**Affiliations:** ^1^Department of Preventive Medicine and Biostatistics, Uniformed Services University of the Health Sciences, Bethesda, MD, United States; ^2^The Henry M. Jackson Foundation for the Advancement of Military Medicine, Inc., Bethesda, MD, United States; ^3^Center for Health Services Research, Uniformed Services University of the Health Sciences, Bethesda, MD, United States; ^4^Department of Medicine, Uniformed Services University of the Health Sciences, Bethesda, MD, United States; ^5^Military Cardiovascular Outcomes Research, Uniformed Services University of the Health Sciences, Bethesda, MD, United States; ^6^James A. Zimble Learning Resource Center, Uniformed Services University of the Health Sciences, Bethesda, MD, United States

**Keywords:** long COVID, PASC, POTS, dysautonomia, treatment, oral medications

## Abstract

**Background:**

Postural Orthostatic Tachycardia Syndrome (POTS) is a complex form of dysautonomia that presents with abnormal autonomic reflexes upon standing, leading to symptoms such as lightheadedness, tachycardia, fatigue, and cognitive impairment. The COVID-19 pandemic has brought renewed attention to POTS due to its overlap with post-acute sequelae of COVID-19 (PASC). Studies have found that a substantial percentage of COVID-19 survivors exhibit symptoms resembling POTS, elevating POTS diagnoses to previously unseen levels. We systematically reviewed the literature for existing high-quality evidence on potential interventions.

**Methods:**

A systematic review of the literature was performed to identify studies of oral medications for the management of POTS. We searched for published manuscripts on the medical management of POTS through 6 April 2024 which met pre-specified inclusion criteria. We conducted quality appraisal and assessed risk of bias before extracting the data and performing synthesis to determine the current state of the evidence; particularly in the context of PASC.

**Results:**

The study search and selection process identified 32 studies that met inclusion criteria, comprising randomized controlled trials, observational studies, and systematic reviews. Most included studies were judged to be of moderate to high quality, with largely low risk of bias. The most frequently studied medications were beta-blockers, ivabradine, and midodrine. Ivabradine and midodrine demonstrated the highest rate of symptomatic improvement, while beta-blockers showed the largest reduction in heart rate variability. Limited evidence was available for PASC-associated POTS, but findings suggest that treatments may have similar efficacy in both PASC and non-PASC cases.

**Conclusion:**

Ivabradine, midodrine, and beta-blockers currently appear to be reasonable front-line choices in pharmacologic management of POTS (PASC associated and otherwise). Further RCTs that evaluate long term outcomes of medications are needed to further establish evidence based pharmacologic treatment approaches for POTS. Particular areas of inquiry include differential efficacy of recommended therapies based on POTS subtypes, and a need for treatments directly targeting the underlying autonomic nervous system dysfunction.

**Systematic review registration:**

PROSPERO, identifier CRD42024505967, https://www.crd.york.ac.uk/prospero/display_record.php?RecordID=505967.

## Introduction

Postural Orthostatic Tachycardia Syndrome (POTS) is a form of dysautonomia characterized by an increase in the heart rate upon standing without orthostatic hypotension (1). The acute rise in heart rate is typically associated with primary symptoms of orthostatic intolerance to include lightheadedness, tachycardia, palpitations, and chest pain with some patients reporting syncope (2). Chronic features of POTS include fatigue, deconditioning, comorbid psychiatric concerns, medical expenditures, and reduced physical, occupational and social functioning (3, 4). If is estimated that POTS may affect up to 1% of the population and it has become increasingly diagnosed in recent years (5). Traditionally it is seen most frequently in women, with onset most often occurring from adolescence through childbearing age (5). There have been several pathophysiological mechanisms proposed, to include dysfunctions in adrenergic function causing a hyperadrenergic state, inadequate cardiac or cerebral perfusion due to dysfunctions in venous return, and dysfunction in the autonomic nervous system (6). Specific onsets or triggers of this condition have been noted in a majority of cases, most commonly secondary to viral infections, trauma, or childbirth (7). A multi-disciplinary approach to the management of POTS has been the mainstay of treatment, with rehabilitative therapies, psychosocial supports, and medications typically used in conjunction to restore patient function (8, 9). Numerous medications have been trialed in POTS, to include beta-blockers or other heart rate control medications to manage tachycardia, mineralocorticoids to improve perfusion, and others targeted at specific symptom management of POTS. However, no medications have been FDA approved for the treatment of POTS (10).

In the wake of the COVID-19 pandemic, a significant proportion of survivors were noted to have symptoms continuing or developing after their acute infection, termed Post Acute Sequelae of COVID (PASC) (11). One of the most common syndromes of PASC bears striking resemblance to POTS, and in some evaluations of PASC patients up to 79% were noted to meet diagnostic criteria for POTS (12). The overlap in these conditions has drawn significant interest, with questions of whether POTS developing as a syndrome of PASC should be managed similarly to non-PASC associated POTS or not (13, 14).

While there are several reviews present on the medical management of POTS, to this point no systematic review has evaluated the evidence for the use of medications in the setting PASC associated POTS. The objective of this review is to provide an update on the overall state of the evidence for pharmacological management of POTS, and to evaluate differences noted in therapeutic response to specific medications in patients with PASC associated POTS and non-PASC POTS.

## Methods

### Study selection criteria

Studies were eligible for inclusion in our review if they were English language articles that included patients diagnosed with POTS being treated with an oral medication for a period of seven days or longer. Studies that specifically evaluated treatments in POTS patients in the setting of post cardiac ablation or based on failure of multiple first line therapies were excluded. All age ranges of patients were considered. Studies with or without a comparator group were included to include observational (i.e., cohort, case series), randomized controlled trials (RCT), and previous systematic reviews and metanalyzes. Published articles as well as articles available on pre-print servers were eligible for inclusion. Conference abstracts and methods papers were not eligible for inclusion. Studies were excluded if they evaluated medications in animal models, if they included only individual case reports of management of POTS, if the medication was delivered in a route other than oral administration, or if the duration of administration was < 7 days.

### Search strategy

A literature search was conducted in LitCOVID, Web of Science, Ovid ALL EBM Reviews, Embase, and PubMed on 26 APR 2024. A total of 1,675 results were retrieved and 649 duplicates removed, leaving 1,026 articles to review. Literature published from the inception date of each database to the date of search and limited English language were considered for inclusion in the review. A search query was developed in consultation with a reference librarian (RA) to include a combination of keywords and subject headings that fully represented each concept. The full query of the search strategy is included in Supplementary Figure S1. The tool Covidence was used for the management of the review process. Covidence is a web-based collaboration software platform that streamlines the production of systematic and other literature reviews.

### Study selection process

After removal of duplicate articles, two authors (BP and KA) independently performed a screening of all titles and abstracts of identified studies and determined if based on the information provided, they met the criteria for study selection. Any disagreements between authors at this stage were decided upon by a separate author, a cardiologist experienced in the management of POTS (MF). Studies that were selected for full text review underwent screening for inclusion by two independent authors (BP and KA). Any disagreements were discussed with a senior author experienced in performing systematic reviews (TK) to reach a consensus on final inclusion of the study into the review.

### Data extraction

All studies included in the review had relevant study variables extracted independently by two authors (BP and KA). Some administrative study details (i.e., author, year of publication) were extracted through an autonomous process by Covidence, however all data related to study design, population, intervention, or outcomes was manually extracted by the reviewers separately in a standardized fashion. Any discrepancies found in the data extraction between the two reviewers was discussed with a senior author (TK) to reach consensus. The variables extracted included the year of investigation, location of investigation, if PASC associated POTS was evaluated, if there were other specific groups under investigation (i.e., pediatric patients, or only those with hyperadrenergic POTS), the inclusion and exclusion criteria of the study, the enrollment of the study and number/reason for dropouts, the study design, the medication under investigation (including dose, and duration of treatment), the proportion of the study group that was female, the mean age of the study group. Specific outcome variables extracted as available included proportion of the treatment group meeting study criteria for treatment success (and the definition of that success), changes in reports of symptom score tools after treatment, and changes in heart rate variability on positional change testing after treatment.

### Bias assessment

All studies included in the review underwent independent critical appraisal and assessment of bias independently by two authors (BP and KA) utilizing critical appraisal tools from the Joanna Briggs Institute (15–18). Each study was evaluated on a variety of domains relevant to their individual study design on a scale of low, high, or unclear risk of bias. Any disagreements between the reviewers on the risk of bias in any study was discussed with a senior author (TK) to reach consensus. Visualizations of the assessed bias in each individual study and amongst all studies of each type were prepared using the Robvis tool (19).

### Data synthesis

A narrative synthesis was performed of the included studies. Information was segregated by medication under investigation and study design. A table was constructed of administrative data for each study to include information such as study location, funding sources, and authorship. Further tables reporting the outcomes of specific interventions for each study design were constructed. Outcome data was evaluated in terms of single arm analysis for the intervention under study, as well as relative to comparator groups as available. For case-series, cohort, and RCT, interventions with ≥2 studies evaluating their outcomes were included in quantitative outcome analysis with tables reporting results of symptomatic and heart rate response among participants. Interventions which were only trialed in one study, and specific differences in outcomes among subgroups were narratively synthesized. Heterogeneity and sensitivity analyses were performed evaluating differences in outcomes between the subgroups of PASC associated POTS as compared to the overall outcomes for each intervention as the data allowed. Publication bias was assessed using funnel plots of the outcome of symptomatic treatment response. A GRADE approach was used to assess the confidence in the studies following the guidance in the Cochrane Handbook (20). Statistical analyses and funnel plot creation were performed using SAS version 9.4 (SAS Institute Inc.). This review was registered, and the full protocol presented on PROSPERO (CRD42024505967). This review followed the PRIMA report guidelines for systematic review, no funding was received to conduct this research.

## Results

### Study selection and characteristics

**Figure 1:**
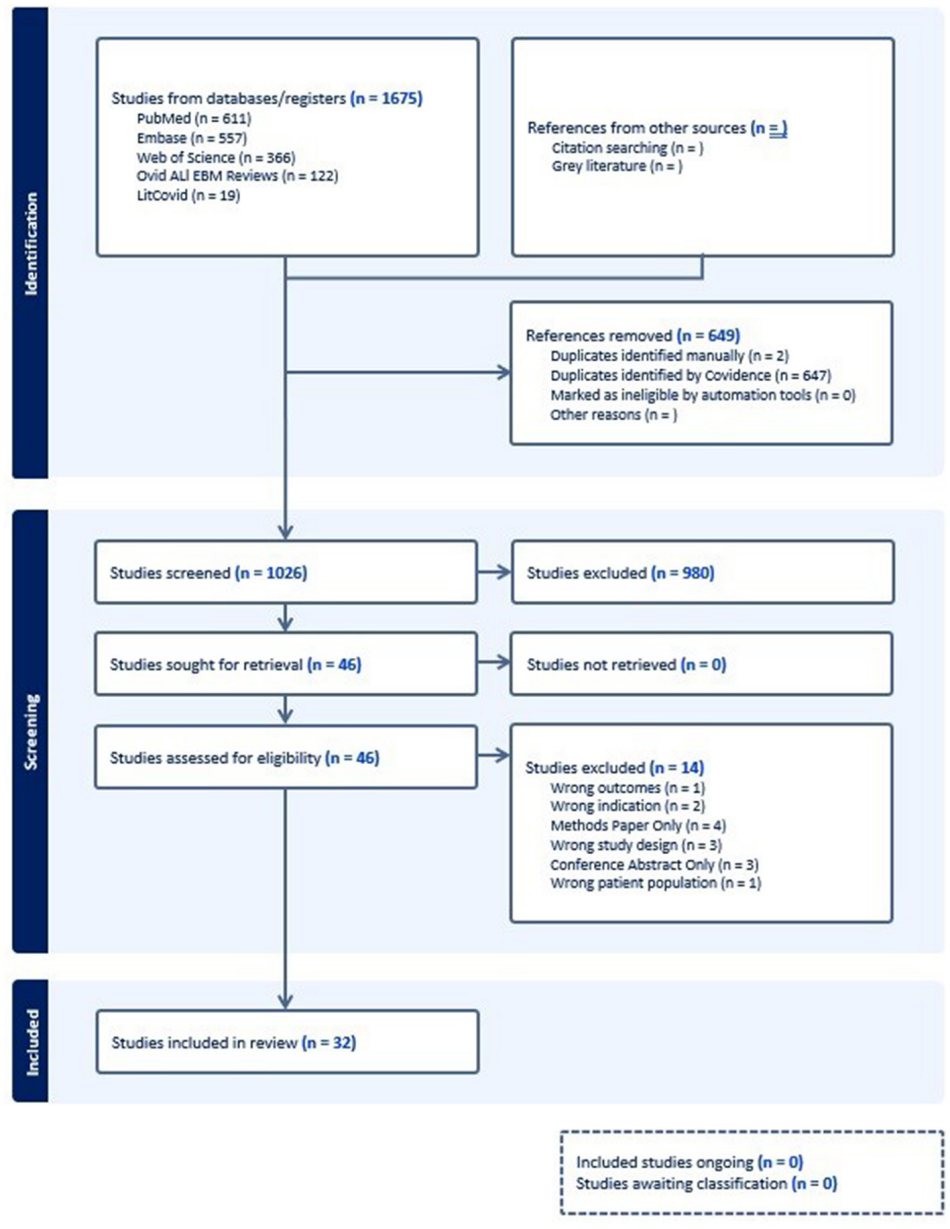
PRISMA Diagram of selected studies.

A total of 1,675 articles were initially identified in the literature search, with 649 identified as duplicates leading to 1,026 articles advancing to title and abstract screening. At this stage 980 articles were found to be irrelevant to the research question and did not meet inclusion criteria for the review. Of the remaining 46 studies undergoing full text review, an additional 14 were excluded, leaving 32 studies remaining in our review. The flowchart of study selection and rationale for study exclusion are presented in Figure 1. The included studies are presented in Table 1, included are 8 case series, 14 cohort studies, 5 RCT, and 5 systematic reviews. Overall, the studies evaluated were generally published recently, with 30 of the 32 identified articles published after 2010. The primary counties in which observational studies and RCT studies were performed were the United States (11) and China (10). The study populations comprised a mix of age ranges, with approximately half (16) of the observational or RCT studies including only children or adolescents in their study population, a smaller number including only adults (2), and the remainder including all age ranges.

**Table 1:** Included studies.

**References**	**Sponsorship source**	**Country**	**Study design**	**End date**	**Start date**	**COVID-19 associated**	**Medications under study**	**Quality assessment**
Taub et al. (41)	A grant from Amgen	United States	RCT	2020	2018	No	Ivabradine	High
Vasavada et al. (43)	None disclosed	-	Systematic review	8-Apr-23	1-Jan-00	No	Midodrine, Desmopressin, Ivabradine, Beta-Blockers, Methylphenidate	High
Stallkamp Tidd et al. (49)	None disclosed	United States	Case series	NR	2023	1 Case	Naltrexone	Moderate
Abdelnabi et al. (36)	None disclosed	United States	Prospective cohort study	NR	NR	Yes	Ivabradine	Moderate
Hasan et al. (44)	The Gregory S. and Elizabeth Wahl Research Fund in Rare, Undiagnosed and Complex Childhood Diseases.	-	Systematic review	11-Feb-20	1999	No	Fludrocortisone, Beta-Blockers, Midodrine, SSRI	High
Towheed et al. (37)	None disclosed	United States	Retrospective cohort study	Feb-19	Jan-15	No	Ivabradine	Moderate
Delle Donne et al. (38)	Clinical Research Unit of the Royal Brompton Hospital.	UK	Case series	Jun-14	Feb-08	No	Ivabradine	Moderate
Gee et al. (45)	None disclosed	-	Systematic review	Aug-17	1956–1957	No	Ivabradine	Moderate
Boris and Bernadzikowski (50)	None disclosed	United States	Case series	Jun-16	Nov-07	No	Methylphenidate, Atomoxetine, Mixed amphetamine salts	Low
Cui et al. (24)	The National High Level Hospital Clinical Research Funding (Multi-center Clinical Research Project of Peking University First Hospital)	China	Retrospective cohort study	Jun-21	Nov-13	No	Metoprolol	Moderate
Wells et al. (46)	National Health and Medical Research Council of Australia	-	Systematic review	May-17	NR	No	Beta-Blockers, Ivabradine, Pyridostigmine	High
Vyas et al. (48)	None disclosed	United States	Case series	2020	NR	No	Bupropion	Moderate
Ruzieh et al. (39)	None disclosed	United States	Case series	Oct-16	Jan-10	No	Ivabradine	Low
Tsuchida et al. (22)	None disclosed	Japan	Case series	May-22	Jan-21	Yes	Bisoprolol	Moderate
Yang et al. (30)	The National Twelfth 5-Year Plan for Science and Technology Support, the Major Basic Research Project of China, and the National Natural Science Foundation of China	China	Prospective cohort study	Feb-12	Jul-11	No	Midodrine	Moderate
McDonald et al. (40)	UK NIHR Biomedical Research Centre in Ageing and Age-related diseases Cardiovascular Theme	UK	Case Series	Jul-10	Jan-08	No	Ivabradine	Low
Boris and Bernadzikowski (31)	None disclosed	United States	Case series	Jun-16	Nov-07	No	Fludrocortisone, Desmopressin, Midodrine	Low
Moon et al. (28)	The National Research Foundation of Korea (NRF) funded by the Ministry of Science, ICT & Future Planning, and Seoul National University Hospital	South Korea	RCT	Aug-15	Apr-14	No	Propranolol, Bisoprolol, Pyridostigmine	Moderate
Deng et al. (47)	The Science and Technology Program of Beijing, Peking University Clinical Scientist Program, and the Fundamental Research Funds for the Central Universities.	-	Systematic review	2019	NR	No	Beta-Blockers	High
Yozgat et al. (42)	None disclosed	Türkiye	Prospective cohort study	NR	NR	No	Propranolol	Low
Wang et al. (25)	The Science and Technology Program of Beijing, Beijing Natural Science Foundation, Peking University Clinical Scientist Program, and the Fundamental Research Funds for the Central Universities.	China	Retrospective cohort study	Jul-19	Nov-10	No	Metoprolol	Moderate
Wang et al. (55)	None disclosed	China	Retrospective cohort study	Sep-19	Jul-12	No	Metoprolol	Moderate
Fu et al. (56)	The National Institutes of Health, National Space Biomedical Research Institute, and the Clinical and Translational Research Center	United States	RCT	2011	NR	No	Metoprolol	High
Chen et al. (29)	The Capital Medical Development Scientific Project, Beijing Science and Technology Plan, and National Natural Science Foundation of China	China	RCT	Jun-10	Oct-07	No	Metoprolol, Midodrine	Moderate
Ross et al. (35)	The National Heart, Lung, and Blood Institute and the Chronic Fatigue and Immune Deficiency Syndrome (CFIDS) Association	United States	RCT	2006	2001	No	Midodrine	High
Liao et al. (32)	The National Twelfth Five-Year Plan for Science & Technology Support of China, the Major Basic Research Project of China, and the Initial Foundation for Youth of Peking University First Hospital	China	Prospective cohort study	Aug-11	Jun-08	No	Midodrine	Moderate
Deng et al. (33)	The Major Basic Research Project of China and the National Twelfth Five-Year Plan for Science & Technology Support	China	Retrospective cohort study	2011	2005	No	Midodrine	Moderate
Zhang et al. (34)	The Major Basic Research Project of China, National Twelfth Five-Year Plan for Science & Technology, Beijing Science and Technology Project, and the National Natural Science Foundation of China	China	Prospective cohort study	2012	NR	No	Midodrine	Moderate
Lin et al. (26)	The National Twelfth Five-Year Plan for Science & Technology Support and Major Basic Research Project of China	China	Prospective cohort study	2015	NR	No	Metoprolol	Moderate
Zhao et al. (27)	The National Twelfth Five-Year Plan for Science & Technology Support, the Major Basic Research Project of China, and from the National Natural Science Foundation of China	China	Prospective cohort study	2014	NR	No	Metoprolol	Moderate
Freitas et al. (23)	None disclosed	Portugal	Prospective cohort study	Dec-98	Jan-97	No	Bisoprolol, Fludrocortisone	Moderate
Lai et al. (21)	Supported by Huseby Family and the American Dysautonomia Institute	United States	Retrospective cohort study	2005	2002	No	Midodrine, Metoprolol, Atenolol	Low

The most common medications evaluated in original research were cardioselective beta-blockers (9 articles) (21–29), midodrine (8 articles) (21, 29–35), ivabradine (6 articles) (36–41), non-cardioselective beta-blockers (2 articles) (28, 42), and fludrocortisone (2 articles) (23, 31), with several other interventions evaluated in one article. Previous review articles have evaluated these agents, as well as pyridostigmine, selective serotonin reuptake inhibitors, and methylphenidate (43–47). Two articles specifically evaluated the treatment of PASC associated POTS, one with ivabradine, and the other with a cardioselective beta-blocker (22, 36).

### Risk of bias and quality assessment

**Figure 2:**
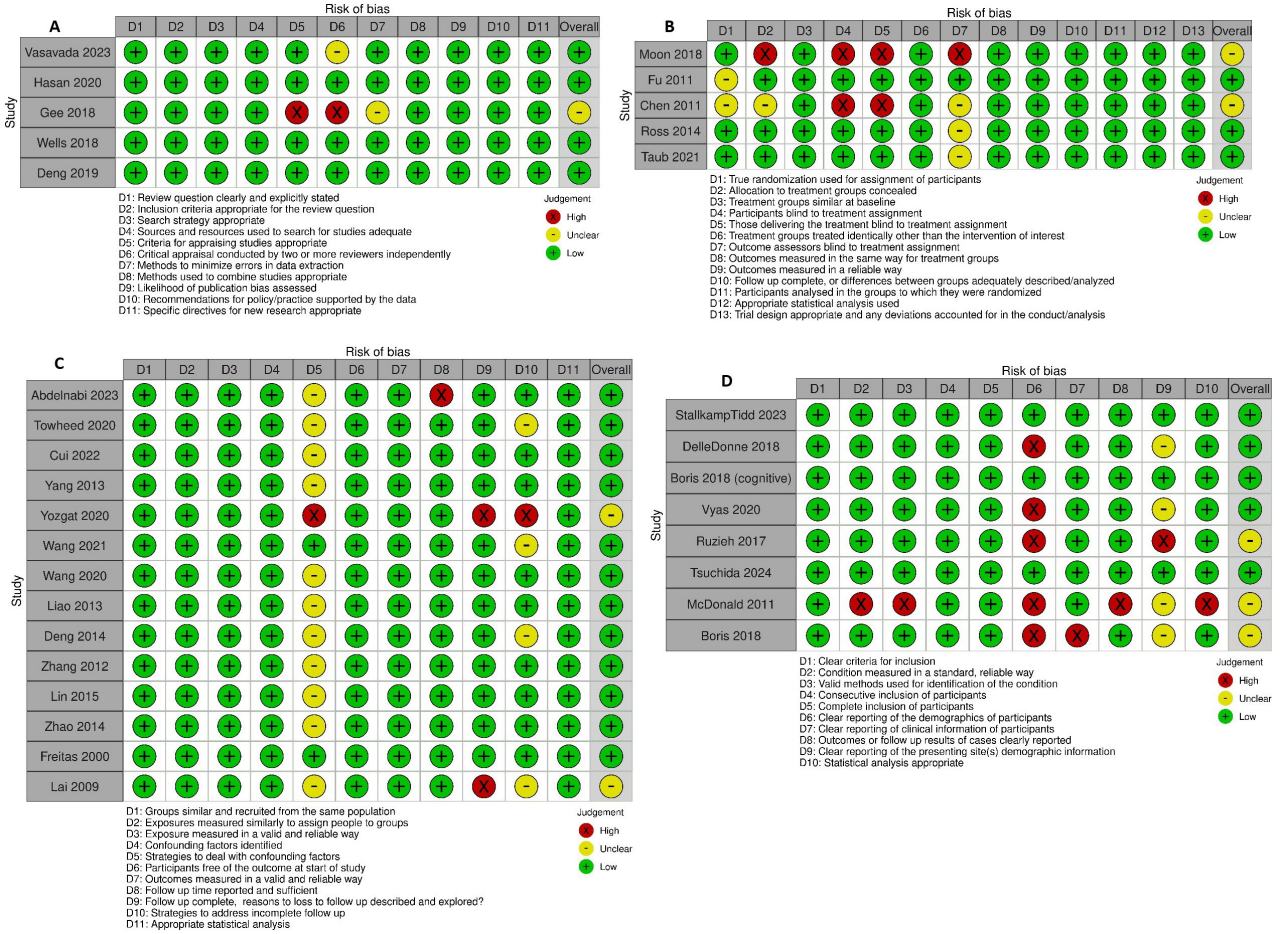
Critical appraisal of studies using Joanna Briggs Institute critical appraisal tools. **(A)** Systematic reviews. **(B)** Randomized controlled trials. **(C)** Cohort studies. **(D)** Case series.

**Figure 3:**
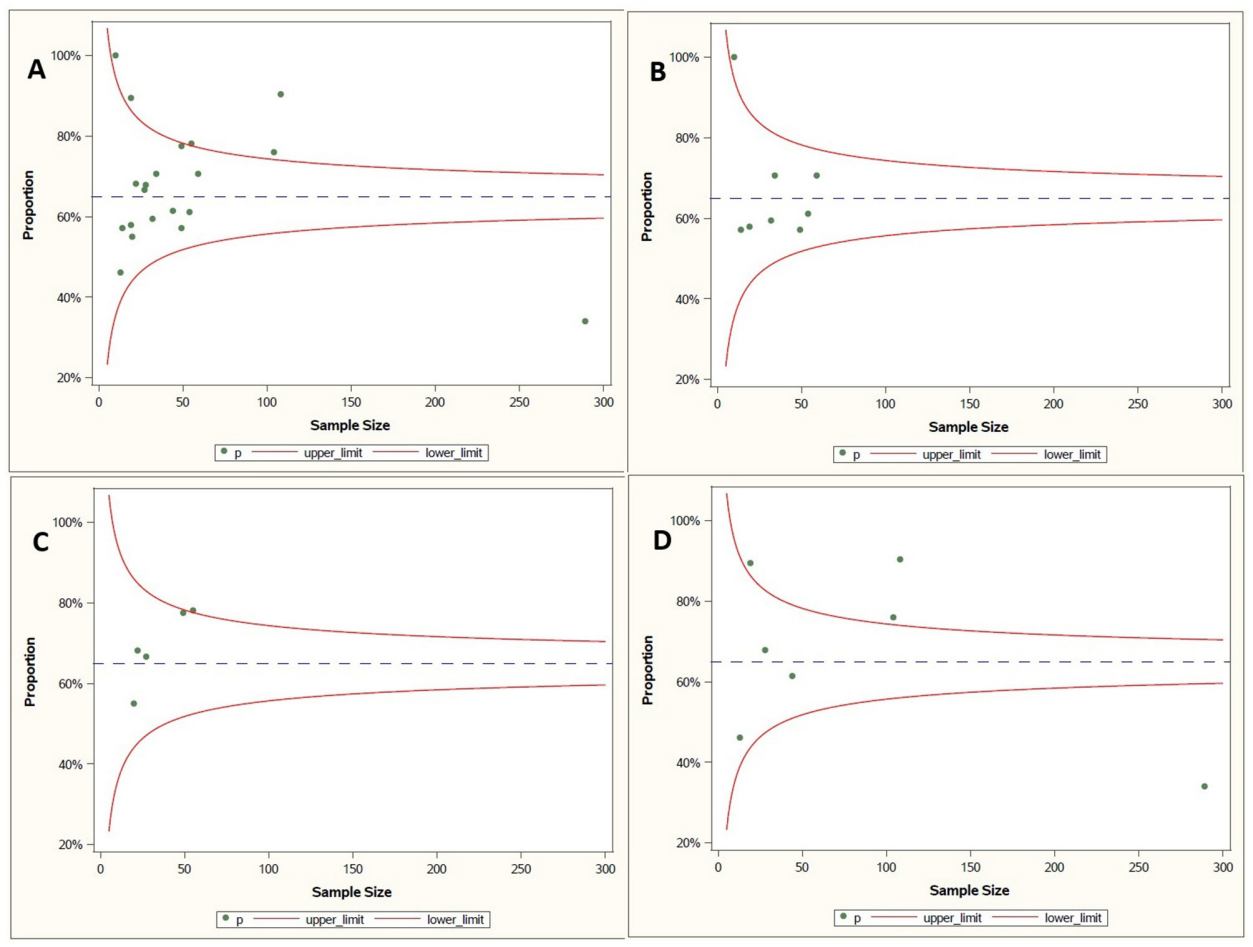
Funnel Plots assessing publication bias for symptomatic treatment effect. **(A)** Overall. **(B)** Beta-blocker. **(C)** Ivabradine. **(D)** Midodrine.

The assessment of bias risk for each study is presented in Figure 2. Utilizing the critical appraisal bias assessment for each study and incorporating general strengths and weaknesses of study approach, the overall GRADE assessment for each study is included in Table 1. Overall, the majority of studies were found to be generally of high or moderate quality. Some areas of concern highlighted in the quality analysis review included several RCTs with unclear methodology regarding randomization and blinding (28, 29), cohort studies with unclear management of confounding variables and concerns over incomplete follow up (21, 36, 42), case series with incomplete reporting of demographics (31, 38–40, 48), and systematic reviews with incompletely described methodology for critical appraisal and data extraction (45). A synthesis of the overall proportion of studies with specific bias concerns are presented in the Supplementary Figure S2. Funnel plots evaluating for risk of publication bias are presented in Figure 3, with no significant concerns identified overall, or for any of the interventions with ≥5 studies included (midodrine, beta-blockers, and ivabradine).

### Study results

The primary endpoints reviewed as available were the proportion of participants meeting the study criteria for treatment success, and the mean change in heart rate variability upon positional change. As available mean changes in symptom score were sparingly reported as well. The uncontrolled response of each medication trialed in at least two studies is reported in Table 2. Given the significant heterogeneity in study design, treatment duration, and definition of treatment success between studies, interpretation of combinations of these measures must be undertaken cautiously. When reviewing treatment success in terms of patients' symptomatic response (either qualitatively assessed as symptomatic improvement, or quantitatively as having a decrement in symptoms score above some threshold) midodrine and ivabradine have response rates of 77.76% and 74.51% respectively, while beta-blockers have a 64.45% response rate. When performing subgroup analysis by study design, midodrine had a higher response rate in the lone RCT evaluating it (89.47%, binary qualitative symptomatic response outcome) than in cohort studies (77.01%, mix of outcome definitions). Beta-blockers had higher response rates in cohort studies (65.75%, mix of outcome definitions), then in the one case series (59.38%, binary qualitative symptomatic response outcome), and RCT (57.89%, binary qualitative symptomatic response outcome) in which they were evaluated. Ivabradine had similar treatment responses in the case series (74.65%, binary qualitative symptomatic response outcome) and cohort studies (74.39%, binary qualitative symptomatic response outcome) with no RCT evidence evaluating this endpoint. Another variable that appeared to potentially skew symptomatic response was the duration of the study. When comparing studies of maximum duration of at least 6 months to those < 6 months, for all medications longer studies had lower response rates (midodrine 82.87% vs. 71.72%, beta-blockers 65.40% vs. 57.57%, and ivabradine 78.18% vs. 72.45%). Fewer studies reported changes in heart rate variability, however there were striking decreases in the pooled changes seen with both cardioselective [15.7 beats/min (bpm)] and non-cardioselective (24.3 bpm) beta-blockers. Midodrine and ivabradine respectively had pooled changes in heart rate variability of 10.3 bpm and 6.1 bpm. Study subtype analysis did find that RCTs reported greater improvements in heart rate variability than other study types, potentially owing to more rigorous methodologies around consistency in measurement. Additionally, longer study duration tended toward lower response in heart rate variability for ivabradine and beta-blockers, but for midodrine studies at least 6 months in duration reported improved heart rate responses. Other medications evaluated in only one observational study with study endpoint of patient symptomatic improvement were naltrexone, which was evaluated individually in a case series, with 50% of participants reporting symptomatic improvement, and bupropion was evaluated in a case series with 58.3% of participants reporting improvement in orthostatic intolerance (48, 49). Yozgat et al. did not evaluate the proportion of participants reporting successful treatment response, but instead reported mean changes in orthostatic intolerance symptom score between a group receiving conventional therapy and a group receiving a combination of conventional therapy, propranolol, and oral rehydration solution for 3 months of treatment (42). In this study the active group had a mean 1.84 point improvement in symptom score as compared to the conventional therapy group which had a mean improvement of 0.42 points in symptom score (42).

**Table 2:** Studies reporting uncontrolled efficacy of medications for POTS.

**Investigational product**	**Study design**	**% Female**	**Mean age (yr)**	**N**	**Treatment duration**	**Treatment success definition**	**Symptomatic efficacy**	**Change in positional heartrate variability**
**Midodrine**								
Yang et al. (30)	Prospective cohort study	57.1	11.5 (2.5)	28	1.5–7 months	Symptom score decrease by ≥2	67.86%	13.5
Boris and Bernadzikowski (50)	Case series	77.5	15.2	289	5 months	Continued Use of medication	33.91%	-
Liao et al. (32)	Prospective cohort study	55.6	12 (3)	108	3 months	Symptom score decrease by ≥2	90.48%	-
Deng et al. (33)	Retrospective cohort study	55.45	11.92 (2.51)	104	6 months	Symptom score decrease by ≥2	75.96%	-
Zhang et al. (34)	Prospective cohort study	49.1	11.5 (2.6)	44	3 months	Symptom score decrease by ≥2	61.36%	5.3
Lai et al. (21)	Retrospective cohort study	76.9	14.3	13	9–50 months	Reported improvement in symptoms	46.15%	-
Chen et al. (29)	Randomized controlled trial	58.5	12.5 (2.2)	19	3–6 months	Reported improvement in symptoms	89.47%	17
Ross et al. (35)	Randomized controlled trial	75	16.8 (0.85)	20	2 weeks	Not reported	-	10.4
**Cardioselective beta blocker (Metoprolol, Atenolol, or Bisoprolol)**								
Lai et al. (21)	Retrospective cohort study	78.6	15.1	14	9–50 months	Reported improvement in symptoms	57.14%	-
Tsuchida et al. (22)	Case series	50	28	32	159 days	Reported improvement in symptoms	59.38%	-
Freitas et al. (23)	Prospective cohort study	100	31 (11)	10	6 weeks	Reported improvement in symptoms	100.00%	-
Cui et al. (24)	Retrospective cohort study	53.7	12.6 (2.7)	54	3 months	Symptom score reduction of ≥50%	61.11%	-
Wang et al. (25)	Retrospective cohort study	45.1	12.0 (2.2)	59	3 months	Symptom score decrease by ≥2	70.59%	-
Lin et al. (26)	Prospective cohort study	47.1	11.7 (2.0)	34	3 months	Symptom score decrease by ≥2	70.59%	-
Zhao et al. (27)	Prospective cohort study	49	12 (2)	49	1.5–3 months	Symptom score decrease by ≥2	57.14%	11
Moon et al. (28)	Randomized controlled trial	52.9	29.8 (9.9)	25	3 months	Not reported	-	28.4
Chen et al. (29)	Randomized controlled trial	58.5	12.4 (1.9)	19	3–6 months	Reported improvement in symptoms	57.89%	11
**Non-Cardioselective beta blocker (Propranolol)**								
Yozgat et al. (42)	Prospective cohort study	67.6	13.26 (2.55)	34	3 months	Not reported	-	-
Moon et al. (28)	Randomized controlled trial	68.4	39.4 (11.6)	26	3 months	Not reported	-	24.3
Abdelnabi et al. (36)	Prospective cohort study	41.8	30.5 (6.9)	55	7 days	Reported improvement in symptoms	78.18%	-
Towheed et al. (37)	Retrospective cohort study	92.6	17	27	3–12 months	Reported improvement in symptoms	66.67%	3.1
Delle Donne et al. (38)	Case series	68.2	14.8 (1.6)	22	0.9–17 months	Reported improvement in symptoms	68.18%	-
Ruzieh et al. (39)	Case series	95.9	35.1 (10.35)	49	3–12 months	Reported improvement in symptoms	77.55%	6.7
McDonald et al. (40)	Case series	83.3	35 (9.9)	20	7–113 weeks	Continued on medication at end of study period	55.00%	-
Taub et al. (41)	Randomized controlled trial	95.5	32.5 (11.4)	22	1 month	Not reported	-	8.3
**Fludrocortisone**								
Boris and Bernadzikowski (50)	Case series	77.5	15.2	582	5 months	Continued use of medication	42.78%	-
Freitas et al. (23)	Prospective cohort study	100	100	1	6 weeks	Reported improvement in symptoms	100.00%	-

When considering other treatment response outcomes, Boris et al published two retrospective analyses of prescription data for POTS patients, one focusing on fatigue and other cognitive symptoms, and one evaluating physical symptoms including orthostatic intolerance (31, 50). Treatment success was defined as repeated prescription of the medication at least 5 times. McDonald et al. used a similar approach in evaluating the treatment efficacy of ivabradine, evaluating whether medication was continued at the end of an observational period (40). In general these methodologies reported lower proportions of treatment success when compared to studies using patient reported outcomes. McDonald reported a 55% treatment success for ivabradine, while Boris found a 33.91% success for midodrine, and 42.78% success for fludrocortisone. Other medications evaluated by this methodology include atomoxetine (16.5%), desmopressin (38.9%), methylphenidate (51.2%), mixed amphetamine salts (44.9%), and modafinil (43.6%) (31, 50).

Table 3 presents comparisons of study endpoints in placebo-controlled studies. Midodrine had slightly greater performance over placebo compared to metoprolol in treatment success and reduction in heart rate variability, while use ivabradine had striking improvements in SF-36 physical functioning scores compared to placebo (29, 35, 41). Table 4 presents comparisons of study endpoints in a study using active comparators. Endpoints were largely similar between bisoprolol and propranolol but adding pyridostigmine to these agents did not significantly improve symptom scores (28).

**Table 3:** Studies reported efficacy of medications for POTS against a placebo comparator.

**Investigational product**	**Study design**	**% Female**	**Mean age (yr)**	**N**	**Treatment duration**	**Treatment success definition**	**Treatment success ratio**	**Placebo**	**Change in positional heartrate variability**	**Placebo**	**Symptom score type**	**Change in symptom score**	**Placebo**
**Midodrine**													
Chen et al. (29)	Randomized controlled trial	58.5	12.5 (2.2)	19	3–6 months	Reported improvement in symptoms	0.89	0.53	17	7	Symptom score	4.1	1.3
Ross et al. (35)	Randomized controlled trial	75	16.8 (0.85)	8	2 weeks	-	-	-	10.4	4.4	-	-	26
**Metoprolol**													
Chen et al. (29)	Randomized controlled trial	58.5	12.4 (1.9)	19	3–6 months	Reported improvement in symptoms	0.58	0.53	11	7	Symptom score	2.2	1.3
**Ivabradine**													
Taub et al. (41)	Randomized controlled trial	95.5	32.5 (11.4)	22	1 month	-	-	-	12.8	4.4	SF-36 Physical functioning	11.8	2.5

**Table 4:** Studies reported efficacy of medications for POTS against an active comparator.

**References**	**Investigational product**	**Study design**	**% Female**	**Mean age (yr)**	**N**	**Treatment duration**	**Change in positional heartrate variability**	**Symptom score type**	**Change in symptom score**
Moon et al. (28)	Bisoprolol	Randomized controlled trial	52.9	29.8 (9.9)	25	3 months	28.4	OIQ	−10.9
Moon et al. (28)	Bisoprolol and pyridostigmine	Randomized controlled trial	60.9	30.3 (14.0)	26	3 months	25.8	OIQ	−10
Moon et al. (28)	Propranolol	Randomized controlled trial	68.4	39.4 (11.6)	26	3 months	24.3	OIQ	−12
Moon et al. (28)	Propranolol and pyridostigmine	Randomized controlled trial	83.3	32.8 (12.8)	16	3 months	24	OIQ	−10.1

Table 5 presents the findings of previous systematic reviews on a study level, while Table 6 presents the findings of previous systematic reviews with participant level results. The findings of previous systematic reviews generally appear to be in line with the findings from the original research identified in this review. Negative findings for pyridostigmine and fludrocortisone as compared to other interventions under investigation are striking, with our review failing to find a positive study of fludrocortisone and another finding symptomatic improvement in only 51% of patients using pyridostigmine (44, 46).

**Table 5:** Review articles reporting results at study level.

**References**	**Group name**	**N (studies)**	**Symptomatic response-reported name**	**Proportion of positive studies**
**Controlled studies**				
Vasavada et al. (43)	Midodrine	4	Positive study (against control)	75.00%
Vasavada et al. (43)	Desmopressin	1	Positive study (against control)	100.00%
Vasavada et al. (43)	Ivabradine	3	Positive study (against control)	100.00%
Vasavada et al. (43)	Beta Blocker	5	Positive study (against control)	80.00%
Vasavada et al. (43)	Methylphenidate	1	Positive study (against control)	100.00%
**Uncontrolled studies**				
Hasan et al. (44)	Fludrocortisone	1	Studies reporting improvement	0.00%
Hasan et al. (44)	B blockers	4	Studies reporting improvement	100.00%
Hasan et al. (44)	Midodrine	3	Studies reporting improvement	100.00%
Hasan et al. (44)	SSRI	1	Studies reporting improvement	100.00%

**Table 6:** Review articles reporting results at participant level.

**Study identifier**	**Number of participants identified**	**Treatment success definition**	**Proportion with treatment success**
**Ivabradine**			
Gee et al. (45)	130	Symptomatic improvement	75.38%
Wells et al. (46)	45	Symptomatic improvement	64.44%
**Overall**			**72.57%**
**Cardio selective beta blockers**			
Deng et al. (47)	249	Symptomatic improvement	79.52%
Wells et al. (46)	151	Symptomatic improvement	60.93%
**Overall**			**72.50%**
**Non-cardio selective beta blockers**			
Wells et al. (46)	16	Symptomatic improvement	**68.75%**
**Pyridostigmine**			
Wells et al. (46)	168	Symptomatic improvement	**51.19%**

Bolded numbers represent overall proportion of all patients with treatment success for all particular oral medication.

Studies where PASC associated POTS was evaluated are presented in Table 7, with comparison of outcomes of studies reporting participant symptomatic improvement in which patients were identified as having PASC associated POTS to the pooled effect of studies that did not evaluate the treatment in the setting of PASC. Ivabradine slightly outperformed its historical use (78.2% of participants meeting study criteria for successful symptomatic improvement vs. 72.5%, while bisoprolol underperformed the historical performance of beta blockers in general (59.4% vs. 65.1%) (22, 36).

**Table 7:** Results of studies evaluating PASC associated POTS as compared to pooled effects in other studies.

	**Treatment Success**
**Ivabradine**	
Abdelnabi et al. (36)	78.18%
Pooled non-COVID studies	72.45%
**B-Blocker**	
Tsuchida et al. (22)	59.38%
Pooled non-COVID studies	65.12%

## Discussion

Our review updates and expands on previous reviews of medical management of POTS by evaluating PASC associated POTS. The most studied medications include midodrine, beta-blockers, and ivabradine. A higher proportion of patients on ivabradine and midodrine reported symptomatic improvement while those on beta-blockers had larger improvements in heart rate variability. Further effects were seen in that studies which followed participants for longer than 6 months tended to see less improvement in patients than those that followed participants for < 6 months. Differing methodologies in assessing treatment success (i.e., patient-based vs. medication continuation) also often had significant heterogeneity in treatment success. Limited studies are available evaluating the efficacy of medical management of PASC associated POTS. However, in those available, treatment results for the most part did not differ greatly from historical treatment efficacy (i.e., non-PASC associated POTS). Ivabradine outperformed historical levels, while bisoprolol underperformed (22, 36). These findings suggest that current medication options for PASC associated POTS are safe and effective, though evidence from randomized trials remains limited. In general, there remains a dearth of randomized controlled studies evaluating the long-term medical management of POTS. Most RCTs have employed control, and often used crossover study design with relatively short treatment periods rather than longer parallel group designs.

There are multiple limitations to the evidence included in this review. Several of the RCTs had unclear methodological reporting regarding the randomization and blinding process, limiting confidence in the results. Additionally, many of the included case reports lacked detailed demographics of the source population and study population in their reviews, potentially limiting the confidence in generalizability. Finally, several of the studies identified in this review came from a single center; raising concerns that any institutional biases present in the performance of research at this center may be overweighted in our review. The studies at this center were noted to report a larger proportion of males compared to most other studies (26, 27, 30, 32–34).

Limitations also exist in the methodology of this review. A key limitation is the lack of consideration for differing adjunct or rehabilitative therapies in treatment of POTS. While the mainstay of POTS treatment is multi modal, often including mechanical device such as compression garments to improve blood flow, management of volume through use of salt loading or structured water consumption, behavioral therapies to identify and avoid symptomatic triggers, and rehabilitative therapies to restore physical and occupational function. While the vast majority of articles included some verbiage around participants continuing to receive standard conventional therapy in addition to medical management, it is difficult to know if standardized approaches to these adjunct therapies were utilized between studies. Medication responses may be confounded by this lack of standardization of adjunct therapies. Additionally limiting the study selection criteria to oral medications taken for >7 days, potentially does not allow for evaluations of injectable medications or regular infusions of medications, some of which are used in the setting of POTS but generally discouraged by treatment guidelines. A final limitation in the methodology of the review design was the inclusion of only English language which may limit the scope and capture of articles that would otherwise meet review inclusion criteria and add to the body of evidence under review herein.

General limitations in the emerging field of dysautonomia therapeutic development include a lack of standardized symptom scoring, primary endpoints or treatment success definitions between studies. While a substantial number of studies used patient-reported improvement as a standard for treatment success, many studies used more quantitative definitions of specific changes in symptomatic score, while other studies defined success by continued use of the medication. Furthermore, multiple symptom scoring systems were used including the Orthostatic Intolerance Questionnaire (OIQ), the 36-Item Short Form Survey Instrument (SF-36), or proprietary scoring systems. This lack of standardization in evaluating symptomology and treatment success impairs the interpretation of the pooled outcomes of the studies. Further, several identified studies did not report treatment endpoints in one or either of the primary domains under consideration (binary treatment success or change in heart rate variability). Outcomes of these studies were narratively synthesized to provide context to how their results added to the body of evidence but were otherwise unable to be compared to other studies in a standardized fashion. In general, there have been few long-term RCTs evaluating medical management of POTS patients. Prior to COVID, the condition was not as commonly recognized, making funding and conducting studies challenging. To date, most pharmacotherapeutic approaches have focused on modulating autonomic dysfunction, rather than attempting to cure or treat the underlying cause. There has also been recent recognition that POTS is not a homogenous diagnosis, with multiple subtypes to include hyperadrenergic, neurogenic and hypovolemic forms now characterized (51). For the most part, studies to date have not attempted to subclassify POTS with a few notable exceptions (41). Further complicating the picture are the variety of mechanistic approaches to treatment with some agents targeting specific symptoms (i.e., naltrexone for fatigue/pain, methylphenidate, or amphetamines for neurocognitive symptoms) while others attempt to intervene mechanistically on heart rate (beta blockers, ivabradine) or venous return (desmopressin, midodrine). Additionally, several of these medications are prescribed off label for the management of POTS, including ivabradine, further complicating the ability of certain patients to receive these medications in some of the studies identified based upon insurance status.

The findings of this review are in line with historical evidence of the medical management of POTS, as evidenced by the similarities in our analysis of original research as compared to the findings of previous review articles included in our analysis. The medications with the most positive evidence supporting their use appear to be midodrine (78% of patients meeting study criteria for successful improvement in symptoms), ivabradine (75%), and beta blockers (64%). At least two randomized trials are currently in progress evaluating ivabradine and IVIG for POTS (52, 53). Limited controlled evidence does not appear to support the use of fludrocortisone or pyridostigmine as first line treatments in the management of POTS, and use of pyridostigmine as an adjunct to beta-blockers also lacks supporting evidence. To this point there have been limited studies evaluating the treatment of PASC associated POTS, with only one randomized study each evaluating ivabradine and bisoprolol found in this review. Further research into the medical management of POTS would ideally include studies of extended duration to establish long term benefit of medications utilized. As there are several medications already routinely used in POTS, active comparators could reasonably be used to simultaneously evaluate comparable benefit of separate medications. Additionally, utilization of adaptive trial designs may allow for the study of more interventions, and combination of interventions in an efficient manner. Studies should systematically and rigorously evaluate these treatments in a prospective fashion with an emphasis on patient centered outcomes, including symptomatic response, social and physical functioning, and other quality of life metrics remain a priority to establishing more evidence-based approaches to the approach to the medical management of POTS patients. Further research on subtyping POTS diagnoses and treatment approach based on individual patient pathological mechanism (hyperadrenergic, hypovolemic, and neurogenic) will provide further evidence to better design studies, optimize diagnosis and treatment methods incorporating relevant advances in the field including the use of wearable technologies and multimodal treatment approaches (54). Special attention should be given to PASC associated POTS both in evaluation and treatment to further elucidate any differences between PASC associated and non-PASC POTS. Defining effective treatment approaches for POTS remains a vital area of research to improve quality of life and function in these patients, and is of growing importance in the wake of the increased recognition of POTS in the wake of the COVID-19 pandemic.

## Data Availability

The original contributions presented in the study are included in the article/Supplementary material, further inquiries can be directed to the corresponding author.

## Author disclaimer

The contents of this publication are the sole responsibility of the authors and do not necessarily reflect the views, assertions, opinions or policies of the Uniformed Services University of the Health Sciences (USUHS), the Henry M. Jackson Foundation for the Advancement of Military Medicine, Inc. (HJF), the Department of Defense (DoD), or the Departments of the Army, Navy, or Air Force. Mention of trade names, commercial products, or organizations does not imply endorsement by the U.S. Government.

## Supplementary material

Supplementary Figure S1 Full search strategy.

Supplementary Figure S2 Overall critical appraisal assessment using Joanna Briggs Institute critical appraisal tools by domain. **(A)** Systematic reviews. **(B)** Randomized controlled trials. **(C)** Cohort studies. **(D)** Case series.
