# Supplementary figures and images for: Oral medications for the treatment of postural orthostatic tachycardia syndrome; a systematic review of studies before and during the COVID-19 pandemic
Source: Front Neurol. 2025 Jan 15;15:1515486. doi: 10.3389/fneur.2024.1515486 (PMC11775448; doi:10.3389/fneur.2024.1515486)

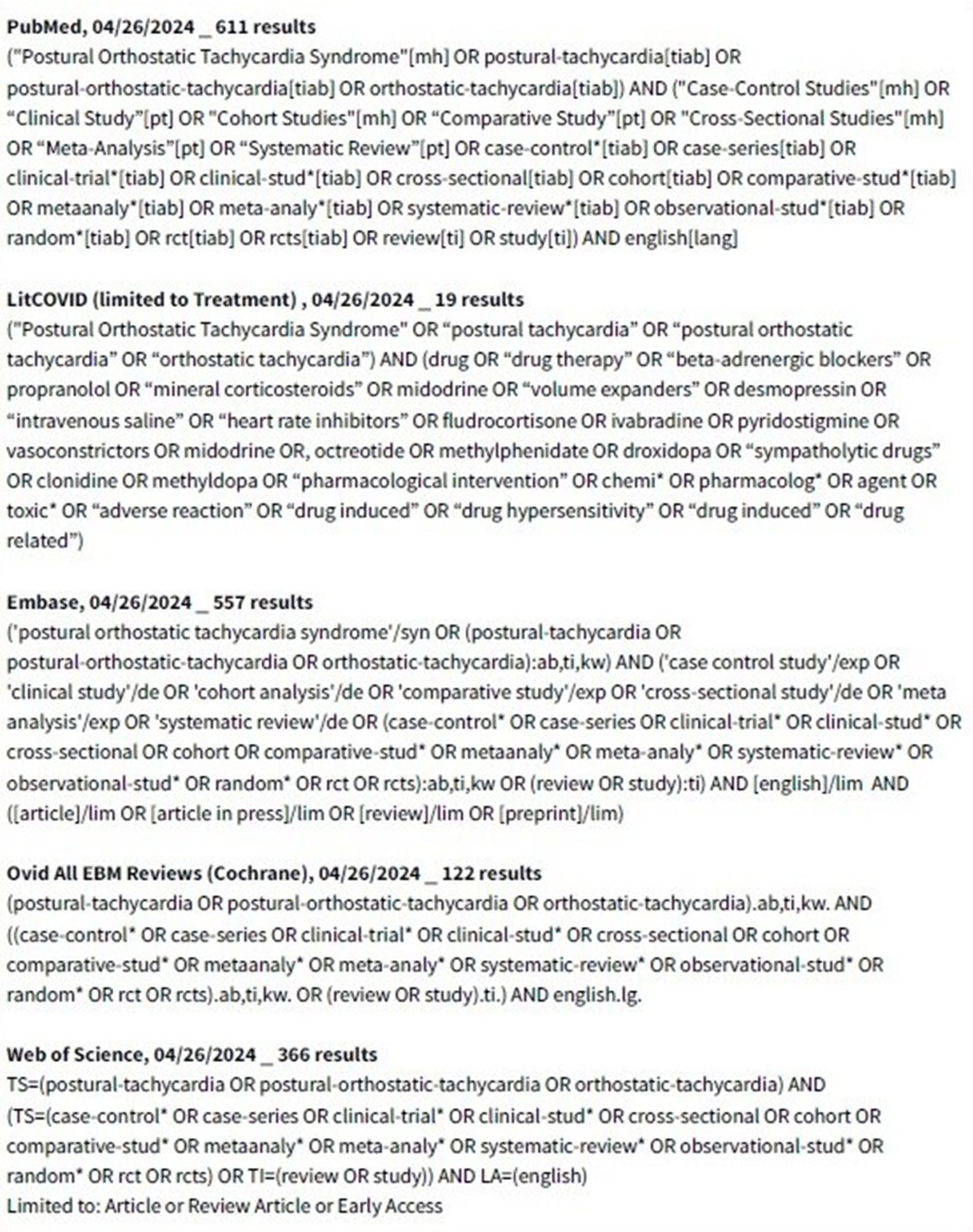

Supplement: Supplementary Figure S1 — Full search strategy. [file Image_1.jpg]

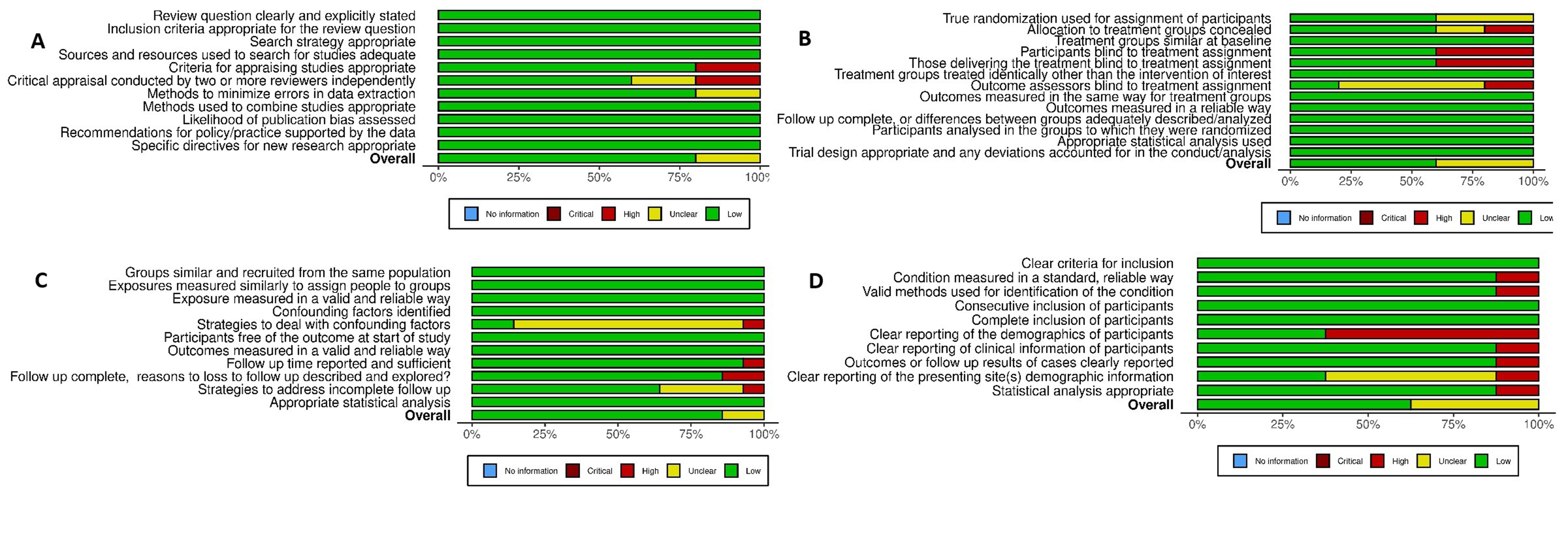

Supplement: Supplementary Figure S2 — Overall critical appraisal assessment using Joanna Briggs Institute critical appraisal tools by domain. (A) Systematic reviews. (B) Randomized controlled trials. (C) Cohort studies. (D) Case series. [file Image_2.jpg]
