# Supplementary material for: Oral medications for the treatment of postural orthostatic tachycardia syndrome; a systematic review of studies before and during the COVID-19 pandemic
Source: Front Neurol. 2025 Jan 15;15:1515486. doi: 10.3389/fneur.2024.1515486 (PMC11775448; doi:10.3389/fneur.2024.1515486)
Supplement: Supplementary file 3 [file Image_3.pdf]

57. Garland EM, Celedonio JE, Nwazue V, Paranjape SY, Black BK, Diedrich A, et al. Carbidopa fails to decrease urinary sodium excretion or improve orthostatic tachycardia in postural tachycardia syndrome. (2016) 26:356

58. Go S, Takahashi R, Suzuki S, Kasuga A, Morichi S, Ishida Y, et al. Retrospective observation of children with the diagnosis of postural tachycardia syndrome while on atomoxetine. Clin Auton Res. (2020) 30:351–3. doi: 10.1007/s10286-020-00701-5

59. Boris JR, McClain ZBR, Bernadzikowski T. Clinical course of transgender adolescents with complicated postural orthostatic tachycardia syndrome undergoing hormonal therapy in gender transition: a case series. Transgend Health. (2019) 4:331– 4. doi: 10.1089/trgh.2019.0041

60. Xu WR, Jin HF, Du JB. Pathogenesis and individualized treatment for postural tachycardia syndrome in children. Chin Med J (Engl). (2016) 129:2241– 5. doi: 10.4103/0366-6999.189915

61. Khurana RK. A double-blind, placebo-controlled, crossover pilot trial of gabapentin for treatment of postural tachycardia symptoms. 30TH INTERNATIONAL SYMPOSIUM ON THE AUTONOMIC NERVOUS SYSTEM. Clin Auton Res 29, 479–546 (2019). https://doi-org.usu01.idm.oclc.org/10.1007/s10286-019-00631-x

62. Peng Y, Wang S, Zou R, Cai H, Zhang J, Wang Y, et al. The influence of sex on the treatment of postural tachycardia syndrome in children. Med Baltim. (2023) 102:e33951. doi: 10.1097/MD.0000000000033951

63. Moon J, Lim JA, Kim TJ, Jun JS, Lee ST, Jun KH, et al. Therapeutic efficacy of propranolol, bisoprolol, and pyridostigmine for postural tachycardia syndrome. 142nd Annual Meeting of the American Neurological Association. *Ann. Neurol.* 82, (2017) 82:S102.

64. Crossover Study of Propranolol vs Ivabradine in POTS. ClinicalTrials.gov identifier: NCT04186286. Last updated May 09, 2024. Accessed October 23, 2024. https://clinicaltrials.gov/study/NCT04186286

65. Ruzieh M, Dasa O, Pacenta A, Karabin B, Grubb B. Droxidopa in the treatment of postural orthostatic tachycardia syndrome. Am J Ther. (2017) 24:e157–61. doi: 10.1097/MJT.0000000000000468

66. Hemodynamic Response of Neuropathic And Non-Neuropathic POTS Patients To Adrenoreceptor Agonist And Antagonist. ClinicalTrials.gov identifier: NCT03070730. Last updated May 18, 2017. Accessed October 23, 2024. https://clinicaltrials.gov/study/NCT03070730

67. ParkerWH,Moudgil R,Wilson RG, Tonelli AR,Mayuga KA, Singh TK. COVID-19 and postural tachycardia syndrome: a case series. Eur Heart J Case Rep. (2021) 5:ytab325. doi: 10.1093/ehjcr/ytab325

68. Efficacy and Safety Study of Efgartigimod in Adults with Post-COVID-19 POTS (POTS). ClinicalTrials.gov identifier: NCT05633407. Last updated October 03, 2024. Accessed October 23, 2024. https://clinicaltrials.gov/study/NCT05633407

69. Campbell IW, Ewing DJ, Clarke BF. 9-Alpha-fluorohydrocortisone in the treatment of postural hypotension in diabetic autonomic neuropathy. Diabetes. (1975) 24:381–4. doi: 10.2337/diabetes.24.4.381
