# Supplementary material for: Oral medications for the treatment of postural orthostatic tachycardia syndrome; a systematic review of studies before and during the COVID-19 pandemic
Source: Front Neurol. 2025 Jan 15;15:1515486. doi: 10.3389/fneur.2024.1515486 (PMC11775448; doi:10.3389/fneur.2024.1515486)
Supplement: Supplementary file 4 [file Table_1.pdf]

Table S1: Table of Studies excluded at full text review.

Study ID	Title	Reason for Exclusion
Garland 2016(57)	Carbidopa fails to decrease urinary sodium excretion or improve orthostatic tachycardia in postural tachycardia syndrome	Conference Abstract Only
Go 2020(58)	Retrospective observation of children with the diagnosis of postural tachycardia syndrome while on atomoxetine	Wrong indication
Boris 2019(59)	Clinical Course of Transgender Adolescents with Complicated Postural Orthostatic Tachycardia Syndrome Undergoing Hormonal Therapy in Gender Transition: A Case Series	Wrong study design
Xu 2016(60)	Pathogenesis and individualized treatment for postural tachycardia syndrome in children	Wrong study design
Khurana 2019(61)	A double-blind, placebo-controlled, crossover pilot trial of gabapentin for treatment of postural tachycardia symptoms	Conference Abstract Only
Peng 2023(62)	The influence of sex on the treatment of postural tachycardia syndrome in children	Wrong outcomes
Moon 2017(63)	Therapeutic efficacy of propranolol, bisoprolol, and pyridostigmine for postural tachycardia syndrome	Conference Abstract Only
NR 2019(64)	Crossover Study of Propranolol vs Ivabradine in POTS	Methods Paper Only
Ruzieh 2017(65)	Droxidopa in the Treatment of Postural Orthostatic Tachycardia Syndrome	Wrong patient population
NR 2012(66)	Hemodynamic Response of Neuropathic and Non-Neuropathic POTS Patients to Adrenoreceptor Agonist and Antagonist	Methods Paper Only
Parker 2021(67)	COVID-19 and postural tachycardia syndrome: a case series	Wrong study design
NR 2022(68)	Efficacy and Safety Study of Efgartigimod in Adults with Post-COVID-19 POTS	Methods Paper Only
Campbell 1975(69)	9-Alpha-fluorohydrocortisone in the treatment of postural hypotension in diabetic autonomic neuropathy.	Wrong indication
Saunders 2023(53)	Comparative Cohort Study of Post-Acute Covid-19 Infection with a Nested, Randomized Controlled Trial of Ivabradine for Those with Postural Orthostatic Tachycardia Syndrome (The COVIVA Study)	Methods Paper Only
